# Re-evaluating the environmental impacts of tourism: does EKC exist?

**DOI:** 10.1007/s11356-019-05269-w

**Published:** 2019-05-09

**Authors:** Jeyhun I. Mikayilov, Shahriyar Mukhtarov, Jeyhun Mammadov, Mayis Azizov

**Affiliations:** 1 Energy Systems and Macroeconomics, King Abdullah Petroleum Studies and Research Center, P.O. Box 88550, Riyadh, 11672 Saudi Arabia; 2 Department of Statistics and Econometrics, Azerbaijan State University of Economics, Istiqlaliyyat Str., 6, AZ1001 Baku, Azerbaijan; 3 Institute for Scientific Research on Economic Reforms, 88a, Hasan Bey Zardabi Avenue, AZ1011 Baku, Azerbaijan; 4 Department of World Economy, Baku Engineering University, AZ0101 Baku, Azerbaijan; 5 Department of Economics and Management, Azerbaijan State University of Economics (UNEC), Istiqlaliyyat Str. 6, AZ1141 Baku, Azerbaijan; 6 Department of Economics and Management, Khazar University, 41 Mehseti Str., AZ1096 Baku, Azerbaijan

**Keywords:** Environmental degradation from tourism, Ecological footprint, Environmental Kuznets curve, Time-varying coefficient cointegration, Resource-rich country

## Abstract

The study investigates the long-run impact of tourism development on ecological footprint by employing the time-varying coefficient cointegration approach (TVC), in addition to the conventional cointegration techniques in the case of Azerbaijan for the period of 1996–2014. Based on the TVC estimation results, the coefficient of tourism development, which is the income elasticity of environmental degradation, was found to be time invariant. The paper uses energy consumption, trade, urbanization, and institutional quality indicators as control explanatory variables. The estimation results revealed that trade and energy consumption have statistically significant and positive impact on ecological footprint, while the coefficients of the other explanatory variables were found to be insignificant. Both the conventional estimation methods and the TVC concluded that, for the relationship between ecological footprint and tourism development, the EKC hypothesis is not present in Azerbaijan. Policy implications for the resource-rich economies have been discussed.

## Introduction

Over the few decades, tourism sector has experienced continuous growth in both the developed and developing countries. Representing one of the main income sources for many developing countries, tourism sector is viewed as an engine of economic growth through the development of infrastructure, the creation of jobs and enterprises, and contribution to the balance of payments. According to the World Tourism Organization (UNWTO), over the last 5 years, international tourism has grown faster than world trade, represented 7% of the world’s exports (generated US$ 1.5 trillion in export earnings) and 30% of service exports, and contributed to 10% of the world GDP in 2016 (UNWTO 2017).

Theoretical and empirical studies suggest that while tourism has a positive contribution to economic growth and development, it is also held responsible for its adverse impacts on the environment. Environmental degradation can occur in two ways. First, as the tourism industry expands, the exploitation of natural resources increases the risk of environmental pollution. More precisely, the tourism industry can cause significant environmental damage in the form of air pollution, natural habitat loss, soil erosion, etc., (Ozturk et al. 2015). Therefore, the tourism sector requires investments into infrastructure such as tourism services (e.g., restaurants, hotels, recreation resorts) and roads, which may help to minimize its adverse effect on the environment. Second, the consumption of energy in the tourism activities such as catering, accommodation, and transportation, lead to higher level of CO_2_ emissions into the environment. Hence, both ways pose threat for sustainable environment and development. Violation of the ecological balance shows itself after time passes. Uncontrolled use of the environment for tourism leads to its destruction and the natural environment is not secured for future generations. Therefore, eliminating the harmful effects of tourism on ecology, protecting nature, as well as for the future development of tourism, is one of the most important challenges. From this point of view, development and promotion of ecotourism are of great importance. The development of this type of tourism can be accompanied by broad financial opportunities and benefits for an economy. Typically, tourists are attracted by ecologically clean regions and countries as they are able to get a beautiful impression of healthy rest and touch with nature. However, ignoring environmental protection can result in the loss of recreational opportunities and tourism potential.

According to the United Nations Environment Program, environmental degradation can be reduced and sustainability can be achieved through the installation of environmentally efficient new technology and establishment of environmental management schemes, which requires direct investments and financial assistance (UNEP 2005). In parallel, the sustainable development requires better institutional quality and the formulation of a legal and regulatory framework for effective and efficient tourism management. As Grossman and Krueger (1995) states, “if the composition of output and the methods of production were immutable, then damage to the environment would be linked unavoidably to the scale of global economic activity.”

This suggests that economic growth promoted by effective regulatory framework is temporarily a remedy to environmental degradation. The environmental degradation and income relationship resembles a relationship between inequality and income relationship described by Simon Kuznets (1955) and later popularized as an environmental Kuznets curve (EKC) by the World Bank Development Report (1992), Grossman and Krueger (1993), and Grossman and Krueger (1995).

Sustainable development or the EKC model hypothesizes that during the initial stage of economic development, a country experiences a degradation of the environment, particularly through deterioration of natural resources that creates environmental impacts such as pollution, soil erosion, and CO2 emissions. But after a certain level of economic growth, as its income increases, the country begins to improve its environmental protection and consequently the level of environmental degradation diminishes. Using panel data analysis of different cities in developed and developing countries, Grossman and Krueger (1995) examined this relationship using various indicators for environmental degradation and found no evidence that environmental quality deteriorates steadily with economic growth. However, for most indicators, economic growth caused an initial stage of deterioration followed by a stage of improvement with varying turning points for different pollutants.

In the recent past, a large number of empirical studies have explored this inverted U–shaped relationship between environmental degradation and per capita income in the context of developed and developing countries, and debatable results have been obtained. These inconclusive results may be due to the fact that GDP, as an indicator for economic growth, is only a rough indicator, does not directly account for environmental quality and capture the effects of different sectors of the economy on environmental degradation (Ozturk et al. 2015; Katircioglu et al. 2018a). As one of the main drivers of environmental degradation, many studies investigated the impact of energy consumption on environmental quality (Kapusuzoglu 2014; Heidari et al. 2015; Anatasia 2015; Kalayci and Koksal 2015; Cetin and Ecevit 2017, inter alia). Although, theoretically, being one of the main drivers, energy consumption negatively affects the environmental quality. However, due to the fact that in practice emissions, like CO_2_, are mainly calculated based on energy consumption. Hence, using energy consumption in the same relationship results in estimation problems, as discussed in Jaforullah and King (2017). In this regard, other drivers of environmental degradation are the focus of the current literature. These indicators might directly influence the environmental quality, or these impacts might be transmitted through other drivers, like energy consumption. In this regard, considering the huge positive impacts of tourism on economic growth and the above-mentioned potential adverse effects on the environment, most recent studies have focused on examining the impact of tourism on environmental degradation in the context of comparative analysis of developed and developing countries (Lee and Brahmasrene 2013; León et al. 2014; Al-Mulali et al. 2014a; Ozturk et al. 2015; Paramati et al. 2017a), Eastern and Western European Union countries (Paramati et al. 2017b), individual countries (Katircioglu 2014a, b; Durbarry and Seetanah 2015), and top 10 tourist destinations (Katircioglu et al. 2018a). In addition, a number of empirical studies concluded that trade and urbanization are other drivers of environmental degradation (Katircioglu et al. 2016; Mikayilov et al. 2017a; Katircioglu et al. 2018c; Katircioglu and Katircioglu 2018, inter alia). Trade impacts environment through importing/exporting emission-intensive goods and services, while urbanization causes through different channels, like increased consumption of energy, impacts through the services and consumption of other necessary goods and services.

To our best knowledge, prior research has not set particular focus on natural resource or oil-rich developing countries which are characterized with their less diversified economies and susceptibility to economic fluctuations due to oil price volatility, which affects the value of national currency and consequently revenues from international economic activities. Therefore, the main objective of resource-dependent countries is to diversify their economies into manufacturing and service industries to protect their economies against the negative impacts of oil price volatility. In this regard, specialized tourism is one of the main priority areas of the development strategy for non-oil sectors such that the tourism industry is considered as an alternative source of foreign currency inflow through the export of services and viewed as an important tool in the improvement of the balance of payments and increase of GDP. Meanwhile, tourism gives a strong impetus to the development and diversification of many areas that serve the tourism industry, and consequently lowers unemployment and contributes to high well-being and poverty reduction.

As a prerequisite for fostering economic growth and ensuring sustainable development, can the economic diversification exert adverse impacts on the environment in resource- or oil-rich countries? To answer this question, we scrutinize this relationship in the context of an oil-rich country, the Republic of Azerbaijan.

The previous researches for Azerbaijan scrutinized the impact of urbanization and population on pollution from transportation (Mikayilov et al. 2017a, b), and the relationship between economic growth and CO2 emissions for the period of 1992–2013 and revealed that the EKC hypothesis does not hold for Azerbaijan (Mikayilov et al. 2018a). However, these studies did not consider the impact of tourism on environmental degradation. In this context, the only research conducted for Azerbaijan was by Ozturk et al. (2015) in a time series analysis for 144 countries for the period of 1988–2008 and concluded the presence of the EKC hypothesis, which we deem not a proper result for Azerbaijan as a developing country especially for a short period of time. In this regard, there is a need for deep analysis in a single country case by employing the time-varying coefficient cointegration approach (TVC), in addition to the conventional cointegration techniques for the period of 1996–2014.

Tourism is one of the booming sectors in Azerbaijan. Although international tourism receipts as a percent of total exports exhibited downward trend until 2006–2008 (1.19% of total exports in 2008), which coincided with the period of high oil rents (% of GDP) that started to decline in 2007, which possibly due to global financial crisis, it started to surge in 2009 and reached to 16.24% of total exports in 2016 (World Bank 2018a, b). Particularly, during 2012–2016, there was a steady increase in the number of entrepreneurship subjects serving in the tourism sector and the number of foreign tourists, numerically being 4.5% and 8.5%, respectively. Meanwhile, the share of tourism industry in the country’s GDP and employment constituted 4.5 and 3.3%, respectively. Particularly, the number of tourism enterprises increased from 96 in 2006 to 272 in 2016 and 339 in 2017, and the number of employees in these enterprises increased 35.7% during 2009–2017 (hotels and related organizations have been excluded) (Strategic Road Map for the Development of Specialized Tourism Industry in Azerbaijan 2016). Hence, the main objective of our research is to explore the Environmental Kuznets Curve hypothesis in the case of an oil-rich developing country, Azerbaijan, by utilizing data on the ecological footprint, international tourism receipts (or GDP from tourism), trade openness, government effectiveness, quality of regulation, and energy consumption for the period of 1996–2014 (the latest data available for the ecological footprint), and also utilize a time series approach, which is the first of its kind in the related literature that takes into account varying nature of the coefficients in the analysis. More specifically, we apply time-varying coefficient cointegration (TVC) approach. Further, we test the validity of the EKC hypothesis and long-run relationships between the ecological footprint and the variables under consideration by using a cointegration technique, autoregressive distributed lag (ARDL) and fully modified ordinary least squares (FMOLS) method. As concluded by some recent studies, such as Liddle and Messinis (2016), Apergis (2016), Moosa (2017), and Mikayilov et al. (2018b), the response of environmental degradation to the change in income variable might have an overtime evolving nature. Therefore, in order to not encounter the spurious regression results if the coefficient is time varying indeed, it is important to model the relationship properly, considering the time variability of coefficient. Furthermore, we advance previous research works by incorporating new explanatory factors such as government effectiveness and quality of regulation as institutional quality indicators. Moreover, our findings will enhance our understanding of tourism management, promote ecotourism, and suggest effective policies for achieving sustainability in Azerbaijan in terms of the environment, tourism, and economic growth.

The remainder of the article is as follows. Section 2 provides a detailed literature review. Section 3 describes the data used and model specification. Section 4 provides the empirical methodology. Section 5 presents the empirical results, and the related discussion is provided in Section 6. Finally, Section 7 provides conclusion and policy implications.

## Literature review

Tourism sector is one of the major contributors of environmental degradation. A proliferation of tourism events is mainly followed by an increased demand for energy for several functions such as transportation, food supplying, housing, and the managing of tourism-related attractions (Becken et al. 2001, 2003; Gössling 2002, 2013; Neto 2003; Becken and Hay 2007; Nepal 2008; Holden 2009; Perch-Nielsen et al. 2010; Howitt et al. 2010; Rosselló-Batle et al. 2010; Dawson et al. 2010; Tsagarakis et al. 2011; Dubois et al. 2011; Becken 2013; Gössling 2013; Tsai et al. 2014; Saenz-de-Miera and Rosselló 2014), which is likely to lead to increased CO_2_ emissions and thus causes environmental degradation (Xuchao et al. 2010; Tovar and Lockwood 2008).

One strand of the literature focuses on the tourism-economic growth nexus. There are many empirical studies, like Balaguer and Cantavella-Jorda (2002), Narayan and Prasad (2003), Dritsakis (2004), Gunduz and Hatemi-J (2005), Katircioglu (2009a, b, c, 2010, 2011), Hye and Khan (2013), Bouzahzah and El Menyari (2013), Jalil et al. (2013), Kilinc et al. (2013), Tang and Abosedra (2014a, b), Al-mulali et al. (2014b), Tang and Tan (2015), Brida et al. (2016), inter alia, investigating this nexus for different countries or country group cases. The second direction in tourism-related literature is the impact of financial development on tourism growth (Katircioglu et al. 2018b, inter alia). The above-mentioned two relationships are not the focus of the current study. Another strand of tourism-related studies is the environmental impacts of tourism activities, which is the focus of our work. There are not many papers in the related literature that investigate the relationship between international tourism and environmental degradation using econometric techniques. Many of these studies used CO_2_ emission as a proxy for the environmental damage. Lee and Brahmasrene (2013) studied the impact of tourism on economic growth and CO_2_ emissions by utilizing panel data for European Union (EU) countries and have found that tourism has a statistically significant negative impact on CO_2_ emissions. Katircioglu (2014a) also examined the impact of tourism development on carbon emissions in Singapore under the framework of environmental Kuznets curve hypothesis for the period of 1971–2010 and employed different time series techniques. The study concluded negative and statistically significant impact of tourist arrivals on carbon dioxide emissions, either in the long- or short-run periods. In addition, the employed Granger causality test concluded unidirectional causality running from tourism development to carbon emissions in the long run. Consequently, the author confirmed the tourism-induced EKC hypothesis in Singapore. Katircioglu (2014b) studied the long-run effects of tourism on carbon dioxide (CO_2_) emissions as a proxy for environmental degradation in Turkey and concluded that tourism growth increases both energy consumption and carbon dioxide emissions. In addition, Katircioglu et al. (2018a) investigated the effect of tourism development on the environmental quality proxied by the ecological footprint in top 10 countries. The estimation results concluded the validity of tourism-induced EKC hypothesis and found negatively significant impact of tourism development on the ecological footprint.

Katircioglu et al. (2014) examined the impact of international tourism on CO_2_ emissions, in the case of Cyprus, a tourist destination island in the Mediterranean region and have concluded that international tourist arrivals have statistically significant positive impact on CO_2_ emissions. The results from Granger causality test showed that tourism development increases carbon dioxide emissions.

Likewise, León et al. (2014) produced the similar findings, i.e., tourism increases CO_2_ emissions, in the case of developed and less developed countries.

Al-Mulali et al. (2014a) studied the relationship between tourism arrivals and CO_2_ emissions from the transportation sector. The study used panel data of 48 top international tourism destinations and came to the conclusion that in all country cases, tourism arrivals significantly increase transportation-related CO_2_ emissions.

Durbarry and Seetanah (2015) investigated the relationship between tourism development and CO_2_ emissions in Mauritius and employed ARDL method and data from 1978 to 2011. The results of the study revealed that an increase in the number of tourists considerably increases CO_2_ emissions.

Dogan et al. (2015) analyzed the long-run relationship between CO_2_ emissions and tourism under the EKC framework in the case of the Organization for Economic Co-operation and Development (OECD) member countries by employing different cointegration tests. The Lagrange multiplier bootstrap panel cointegration test concluded the existence of a long-run co-movement among the variables under study. The dynamic ordinary least squares (DOLS) estimation results indicated that tourism increases CO_2_ emissions.

Using the ecological footprint as a measure of environmental degradation, Ozturk et al. (2015) examined the impact of tourism on the environment and tested the existence of the EKC phenomenon for the case of 144 countries, including Azerbaijan, for the period of 1988–2008. The study employed the GMM method for the time series data and also used the system GMM for the panel data, and added control variables such as energy consumption, trade openness, and urbanization. The estimation results showed that for the upper middle- and high-income countries, the relationship is negative between the ecological footprint and its determinants in most cases. Furthermore, the study concluded that the EKC hypothesis is mainly presented in the upper middle- and high-income countries. Further, authors confirmed the presence of the EKC hypothesis for Azerbaijani case.

The existence of EKC hypothesis was also confirmed by de Vita et al. (2015) for Turkey, and by Zaman et al. (2016) and Paramati et al. (2017a) for developed and developing countries, by Shakouri et al. (2017) for Asia-Pacific countries, and by Alam and Paramati (2016) for 49 developing countries. While Solarin (2014) in the case of Malaysia, Zhang and Gao (2016) in the case of China, Bozkurt et al. (2016) in the case of BRICTS countries, Naradda Gamage et al. (2017) in the case of Sri Lanka, and Dogan (2017) in the case of OECD countries found that EKC hypothesis does not hold for the studied country cases. Raza et al. (2016) used wavelet based analysis to study the impact of tourism development on carbon emissions for the US case. The results showed that tourism development has a statistically negative and significant impact on the environment.

Yorucu (2016) examined the long-run impact of foreign tourist arrivals on CO_2_ emissions. Author utilized ARDL approach and made use of annual data from 1960 to 2010. The paper concluded that there was a long-run causal relationship from the growing number of foreign tourist arrivals toward the growth of CO_2_ emissions.

Paramati et al. (2017b) examined the effect of tourism on economic growth and carbon dioxide emissions for the case of Eastern and Western European Union (EU) countries. Results of the study revealed that, in the case of Eastern EU, tourism increases CO_2_ emissions, while it decreases the emissions in Western EU countries.

Azam et al. (2018) investigated the impact of tourists’ arrivals on environmental pollution (CO_2_ emissions) in Malaysia, Thailand, and Singapore for the period of 1990–2014 by employing FMOLS method. Authors found that tourism has a statistically significant positive impact on environmental pollution in Malaysia while a negative effect was determined in the cases of Thailand and Singapore.

Mikayilov et al. (2018a) investigated the relationship between economic growth and CO_2_ emissions, employing different cointegration methods to time series data of Azerbaijan from 1992 to 2013, and concluded that the EKC hypothesis does not hold for Azerbaijani case. The study uses CO_2_ emissions as a measure of environmental degradation. Mikayilov et al. (2017a, b) examined the impacts of urbanization and population on pollution from transportation and did not study the impact of tourism-related income on environmental degradation.

As can be seen from the literature review, there is only one time series study, Ozturk et al. (2015), devoted to the impact of international tourism on environmental degradation in the case of Azerbaijan, which concluded the presence of the EKC hypothesis. They investigated the case for a relatively old period, namely 1998–2008 and concluded the EKC, which we think is not a proper result for Azerbaijani case, as a developing country. In addition, most of the previous studies examined the nexus between tourism indicators and CO_2_ emissions across the developed and developing economies but did not consider the ecological footprints. Given these limitations and research gaps, the present study aims to examine the effect of international tourism on ecological footprints in the case of Azerbaijan. Hence, our study adds an important value to the empirical literature on the role of international tourism on environmental degradation. Further, to the best of our knowledge, this is the first study, in the tourism-related literature, to employ time-varying coefficient approaches. Therefore, the findings derived from our study will be critical for the policy and practice for the sustainable tourism management in Azerbaijan and similar countries.

## Model specification and data

### Model specification

In this study, we make use of the theoretical approach proposed by Shafik and Bandyopadhyay (1992). Precisely, in our empirical setting, we employ the conventional framework which relates the environmental indicator and its potential determinants. The study makes use of the following cubic functional specification with respect to the income proxy and added some other relevant explanatory variables:

1 $$ ei={b}_0+{b}_1{y}_t+{b}_2{y}_t^2+{b}_3{y}_t^3+{b}_4{x}_t+{\varepsilon}_{\mathrm{t}} $$

where *ei* is environmental indicator, while *y* and *x* are the income and a vector of additional explanatory variables, respectively. Further, *t* and *ε* are the time period and error term, respectively. Since, we aim to investigate the impact of international tourism on environmental degradation, following Ozturk et al. (2015), Katircioglu et al. (2018a), inter alia, we use the *ecological footprint* as a proxy for the environmental degradation. While all other indicators such as CO_2_ emissions, SO_2_ emissions, dark matter, and suspended particle matter represent only a small portion of environmental degradation, as argued by Ozturk et al. (2015). We use *international tourism receipts* as a proxy for the income from tourism sector. To test different hypotheses in line with the general framework, we added the squared and cubic terms of this variable. We also employ and test different variables, considered as drivers of the environmental degradation, such as *total trade*, *energy consumption*, *urbanization*, *government effectiveness*, and *regulatory quality*. All variables, except the last two, are used in logarithmic form.

Further, we argue that in the studies devoted to the impact of tourism sector on environmental indicators and to testing the EKC hypothesis, the model specification needs to be properly set to avoid theoretical and econometric drawbacks such as multicollinearity and redundant variables. Some papers employ *international tourism receipts* and overall *gross domestic product* (GDP) as explanatory variables, but as it is known, the second one includes the first one and consequentially the researcher will be encountered with perfect or near-perfect multicollinearity, and in the best case the estimated coefficients are not reliable. Another issue in the related studies is using *energy consumption* in the specification if the environmental degradation is proxied by CO_2_ emissions, because as known due to the lack of reliable data, CO_2_ emissions are mainly calculated using *energy consumption*. Hence, using *energy consumption* as an explanatory variable in CO_2_ specification will result in the problems discussed in Jaforullah and King (2017) and may lead to unreliable findings. Yet another problem is the misinterpretation of the impact of an explanatory variable on the dependent variable, when the powers of that variable are included in the specification. In this case, the elasticity of the dependent variable with respect to that variable should be calculated and reported as a response of the dependent variable to the change in the mentioned variable.

### Data

We employ annual time series data from 1996 to 2014 for empirical analysis.^1^ The selection of the sample period is purely based on the availability of the data. The variables of the study are measured as follows: ecological footprint (EF) is the sum of the cropland, grazing, fishing, forest, CO_2_ emissions, and infrastructure footprints. To put it differently, it is the area of land and ocean needed to support the countries’ consumption measured in hectares (Ozturk et al. 2015, inter alia). The international tourism receipts (TR) are used as a proxy for tourism and it is measured in million constant US dollars. The total trade of goods and services (TD) is the sum of exports and imports in million constant US dollars. Energy consumption (EC) is measured in kilograms of oil equivalent. The total urban population, in persons, is an indicator of urbanization (UR). Government effectiveness (GE) reflects “perceptions of the quality of public services, the quality of the civil service and the degree of its independence from political pressures, the quality of policy formulation and implementation, and the credibility of the government’s commitment to such policies” (World Bank 2018b). Finally, regulatory quality (RQ) reflects “perceptions of the ability of the government to formulate and implement sound policies and regulations that permit and promote private sector development” (World Bank 2018b). The data for the ecological footprint has been sourced from the Global Footprint Network, while data on international tourism receipts, total trade, energy consumption, government effectiveness, and regulatory quality have been taken from the World Bank. In empirical estimations, all the variables, except the last two, were used in logarithmic form.

**Fig. 1:**
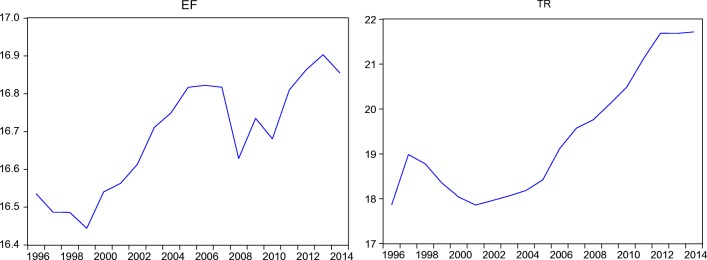
Time profile of the variables (in logarithmic form)

Figure 1 below shows the time-varying trends of ecological footprint and international tourism receipts (the main variables of interest), both in the natural logarithm levels over the period of 1996–2014.

As demonstrated in the Fig. 1, the EF sharply decreased in Azerbaijan for the period of 1996–1999. This decrease can be explained by different factors, such as the shutdown or weakening of the industrial sector after the collapse of the Soviet Union. For the 1999–2014 time span, the relative increase with some volatility can be observed in EF. For the period corresponding to the global financial crisis, 2007–2008, there was a decline in the path of the variable, and it starts to increase with economic recovery process. On the other hand, TR relatively increased during 1996–1997 and decreased in the period of 1998–2001, which is coincided with the beginning of the Russian financial crisis in 1998, which severely affected the economies of neighboring countries. As a general tendency for the chosen period, TR has increased persistently since 2001.

The descriptive statistics of the employed variables are displayed in Table 1. As can be seen from Table 1, comparing with the previous relatively longer periods, volatility in all variables (except GE) exhibits decreasing nature, indicating more stable behavior over time.

**Table 1:** Descriptive statistics of the variables for three time periods

	Mean			Standard deviation			Coefficient of variation, %		
	1996–2014	2005–2014	2010–2014	1996–2014	2005–2014	2010–2014	1996–2014	2005–2014	2010–2014
EF	17.85	19.70	20.28	25.78	16.37	16.75	14	8	8
TR	6.72	11.20	18.68	8.23	9.36	7.41	122	84	40
TD	265.01	416.38	469.84	176.99	83.82	43.72	66	20	9
EC	1451.60	1452.35	1419.57	81.35	102	93.19	6	7	7
UR	4.49	4.78	5.01	0.38	0.27	0.14	9	6	3
GE	−0.79	−0.68	−0.64	0.17	0.15	0.21	22	22	32
RQ	−0.63	−0.41	−0.38	0.29	0.09	0.07	45	23	18

*EF* ecological footprint, in mln hectares; *TR* tourism receipts, in bn constant US dollars; *TD* trade, in billion constant US dollars; *EC* energy consumption, in kg of oil equivalent; *UR* urbanization, in mln persons; *GE* government efficiency; *RQ* regulatory quality

In empirical analyses, we also included a pulse dummy variable to catch the drop in 2008 in the path of ecological footprint.

## Econometric methodology

In this study, we investigate the effect of tourism on the environmental damage (utilizing the ecological footprint) and examine the EKC hypothesis. First, we will check the non-stationarity characteristics of the variables. For this purpose, we use the augmented Dickey-Fuller (ADF, Dickey and Fuller 1981) and Phillips–Perron (PP, Phillips and Perron 1988) unit root tests.

Next, if the variables are integrated on the same order, then we can test whether they move together in the long run, using cointegration tests. For this exercise, we employ the single equation–based cointegration method, which is autoregressive distributed lags bounds testing (ARDL) approach developed by Pesaran and Shin (1999) and Pesaran et al. (2001) as it outperforms all the alternative cointegration methods in small samples, as in our case we only have 19 observations. We also employ FMOLS method (Saikkonen 1992; Stock and Watson 1993), which is based on the residual-based cointegration method developed by Engle and Granger (1987).

Since the above-mentioned unit root tests, ARDL and FMOLS cointegration methods are widely used techniques in similar studies, we do not describe them. For further details, readers can refer to Dickey and Fuller (1981), Phillips and Perron (1988), inter alia, for the ADF test; and Pesaran and Shin (1999), Pesaran et al. (2001), Hansen (1992a, b), Phillips and Hansen (1990), Hamilton (1994), Park (1992), Saikkonen (1992), and Stock and Watson (1993), for the cointegration tests, inter alia.

As a further robustness check, we employ time-varying coefficient cointegration approach (TVC) proposed by Park and Hahn (1999) which has several advantages over the conventional fixed coefficient methods. First of all, consider the fact that the response of the dependent variable to the explanatory variables changes due to different factors such as structural changes and may vary over time, as discussed in Park and Hahn 1999; Chang et al. 2014; Mikayilov et al. 2018b, inter alia. Second, if the true relationship among the variables is time varying, but if it is estimated using the fixed coefficient technique, then the results will be spurious. For further discussion of the TVC approach, see, for example, Park and Hahn (1999), Chang et al. (2014), and Mikayilov et al. (2018b). Third, the existence of the EKC implicitly means variation in the income elasticity of environmental indicator, which is easier to observe employing the technique which takes into account this feature explicitly. To the best of our knowledge, this is the first study to apply TVC techniques in the tourism literature. However, Liddle and Messinis (2016), Apergis (2016), Moosa (2017), and Mikayilov et al. (2018b, c) employed these techniques to investigate the impact of economic development on CO_2_ emissions, while the current study investigates the impact of tourism on ecological footprint, which is a more relevant variable to access the impacts on environmental degradation.

## Empirical results

### Unit root tests results

First, we should check the stationarity properties of the used variables. As mentioned in the methodology section, for this purpose, we use the ADF and PP unit root tests. The results of the unit root tests are presented in Table 2. The unit root tests results reveal that all the variables, except urbanization, are non-stationary at their levels but they are stationary at first differences, being integrated of order one, I(1)*.* It is worth to note that Mukhtarov et al. (2017, 2018) also found energy consumption (EC) variable to be I(1) as a result of different unit root tests for Azerbaijan case. According to the results of the tests, only *urb* is stationary at second difference, being integrated of order two. As discussed in Hasanov et al. (2016) and Mikayilov et al. (2017a, b), the demographic variables such as population and urbanization demonstrate the feature of I(2) variables, but this is not in line with their graphical visualization as well as the common sense regarding the nature of these variables, rather this result can be caused by the small sample size, which is a case in our time period. Hence, as a research decision, we conclude that urbanization variable is also integrated of order one, and proceed to the next step. Since the variables of interest are integrated of the same order, namely they are I(1) variables, we can proceed to the next step, testing the existence of cointegration among the employed variables.

**Table 2:** Results of unit root tests

Variable	The ADF test				The PP test	
	Level	*k*	First difference	*k*	Level	First difference
EF	− 1.038	0	− 4.768***	0	− 1.067	− 4.756***
TR	0.067	0	− 3.040**	0	− 0.242	− 3.056**
TD	− 1.534	1	− 3.195**	0	− 1.069	− 3.153**
EC	− 2.941	0	− 6.786 ***	0	− 2.859	− 3.629 **
URB	0.709	1	− 1.607	0	− 2.731	− 1.873
RQ	− 2.068	0	− 2.803*	0	− 2.291	− 2.772*
GE	− 0.008	0	− 3.871**	0	− 0.008	− 3.871**

ADF and PP denote the augmented Dickey-Fuller and Phillips–Perron tests respectively. Maximum lag order is set to two and optimal lag order (*k*) is selected based on Schwarz criterion in the ADF test; Triple asterisk, double asterisk, and single asterisk indicate rejection of the null hypotheses at the 1%, 5%, and 10% significance levels respectively. The critical values are taken from MacKinnon (1996) for the ADF and PP tests respectively

### Cointegration and long-run estimation results

As a next step, we test the variables for long-run co-movement. We employ Pesaran’s (Pesaran et al. 2001) Bounds test and FMOLS-based Engle-Granger tests for this exercise. First, we used the cubic specification for the ecological footprint function and tested the relationship for the cointegration. The test results revealed that the variables are cointegrated (to save space we do not report the test results but they are available upon the request). After having cointegration relationship among the variables, we can test the specification. The cubic term is found to be insignificant. Hence, based on the procedure proposed by Shafik and Bandyopadhyay (1992), we dropped it. Next, we do the same exercise for the quadratic specification. The results of cointegration tests for quadratic specification are given in Table 3. Both cointegration tests conclude the existence of cointegration relationship among the variables. In other words, the variables move together in the long run. Since we have more than two variables, to robustify the cointegration test results and justify the use of single equation estimation methods, we tested the existence of long-run relationship using Johansen’s trace and rank tests (Johansen 1988; Johansen and Juselius 1990). Both tests concluded one cointegration relationship that is, for the null hypothesis “there is at most one cointegration relationship” test statistics for trace and rank tests are 42.27 and 23.71, while the 5% critical values are 47.86 and 27.58, respectively. That is, we can continue with the single-equation estimation techniques.

**Table 3:** Cointegration tests’ results

Bounds test	Engle-Granger test
19.567***	− 5.682**

Bounds test stands for the Pesaran’s ARDL–based *F* test and Engle-Granger test stands for FMOLS–based Engle-Granger test. Triple asterisk and double asterisk stand for cointegration at 1% and 5% significance level, respectively. Since we have a small sample size in the Bounds test, we used Narayan (2005) critical values

Considering the conclusion that the variables share a common trend in the long run, we can estimate the long-run relationship among them. As mentioned in the methodology section, we employ the ARDL and FMOLS methods first. The long-run estimation results are given in Table 4. We estimated different models to test different hypotheses, namely we estimated 8 different models. The main method is ARDL, since it outperforms its counterparts in the small sample case. Hence, the main model is Model1 in Table 4. As can be seen in the models M3 to M8, we tested the *urbanization*, *government efficiency*, and *regularity quality* to see if urbanization- and government-related administrative quality measures have impact on the environmental quality. As can be seen from Table 4, all three variables were found to be insignificant by either estimation method.

**Table 4:** Long-run estimation results

Indicator	Ecological footprint							
Method	ARDL	FMOLS	ARDL	FMOLS	ARDL	FMOLS	ARDL	FMOLS
Variables/models	M1	M2	M3	M4	M5	M6	M7	M8
td	0.17***	0.19***	0.21***	0.19***	0.22***	0.20***	0.12***	0.18***
tr	− 1.07**	− 0.55***	− 1.17***	− 0.60**	− 0.80***	− 0.56***	− 1.10**	− 0.54***
tr2	0.04**	0.02***	0.04***	0.02**	0.03***	0.02***	0.04**	0.02***
ec	0.55**	0.74***	0.67***	0.74***	0.60***	0.68***	0.71***	0.66***
ge	–	–	–	–	–	–	0.20	0.03
Rq	–	–	–	–	− 0.10	− 0.02	–	–
Urb	–	–	0.16	− 0.11	–	–	–	–

*tr* international tourism receipts, *td* trade, *ec* energy consumption, *ge* government effectiveness, *rq* regulatory quality, *urb* urbanization; Triple asterisk, double asterisk, and single asterisk stand for the rejection of null hypothesis at 1%, 5%, and 10% respectively. Mi’s stand for models 1 through 8

For robustness check, Model 2 is the FMOLS based one. To conclude, our main models are M1 and M2. The results from M2, FMOLS based, are quite close to the M1 (ARDL based) results, which can be seen as a robustness of our results. The closeness of the coefficients through the variables, even after adding new explanatory variables to the model, also can be considered as a robustness of the specification.^2^

### Results of time-varying coefficient cointegration approach

As mentioned before, we employ the TVC approach for further robustness check to avoid the drawbacks discussed by Liddle and Messinis (2016), Apergis (2016), Mikayilov et al. (2018b), inter alia. Since we have a small sample, we use only the income proxy, namely *international tourism receipts*, as an independent variable. Taking into account the effects over time, employing varying coefficient, this approach is not sensitive to the omitted variable bias; hence, we can continue our exercise. First, we test variables for cointegration relationship. Park and Hahn (1999) advocate that the failure to find cointegration relationships in many studies based on fixed-coefficients parametrizations might be the result of parameter instability. Hence, we employ the variable addition test (VAT, Park 1990) to examine if the variables move together in the long run.

The idea of the VAT test is simply to look at the joint statistical significance of the coefficients of the added polynomial trend variables. The results of the VAT cointegration test are given on the left-hand side of Table 5. As can be seen from the table, the test concludes cointegration relationship between the variables at 1% significance level, supporting Park and Hahn’s (1999) argument.

**Table 5:** Results of the test for joint significance of coefficients and cointegration tests

Variable addition test (VAT)				Test for joint significance of time-varying coefficients			
Test statistics	1% CV	5% CV	10% CV	Test statistics	1% CV	5% CV	10% CV
2.35***	13.18	9.49	7.78	2.66	15.09	11.07	9.24

The left-hand side of the table shows the results of VAT cointegration test. The right-hand side of the table demonstrates results of the joint significance test of time-varying coefficients, in order to test whether or not the income elasticity is fixed or time varying. Triple asterisk and double asterisk stand for acceptance of the null in case of VAT test and rejection of the null in Chi-square test at 1% and 5% significance level, respectively

**Fig. 2:**
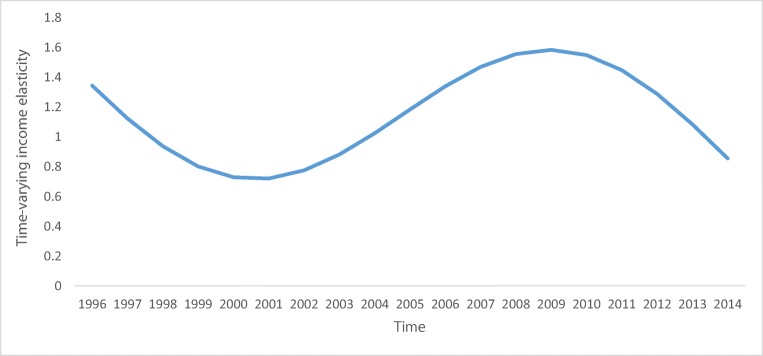
Time-varying income elasticity, versus time

**Fig. 3:**
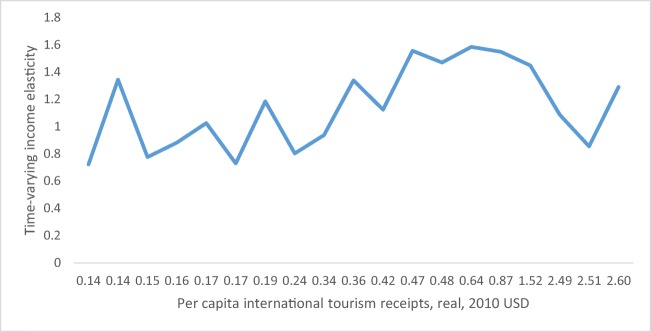
Time-varying income elasticity, versus income

As a next step, we test whether the time-varying coefficient is significant or not. This is the Wald test of joint significance of the varying coefficients (Chang et al. 2014). The test results are given on the right-hand side of Table 4. As can be seen from Table 4, the test concludes the insignificance of the varying coefficient. Despite the fact that the time-varying coefficient is found to be insignificant, meaning that the difference between different values of income elasticity across the time is negligible (insignificant), its time profile says some insights about the developing nature of that country. In addition, the values plotted versus per capita income also enables to conclude the idea about the EKC phenomenon in that country case. We provided these graphs for the above-mentioned purposes. The profile of the time-varying coefficient is given in Fig. 2, against time and Fig. 3, against per capita income levels.

To see the visual exercise for the EKC, Fig. 3 can be observed. At the first glance, as the graph of the varying elasticity shows, the response of *ecological footprint* to *international tourism receipts* does not seem to support the EKC phenomenon.

## Discussion of the results

Starting with the conventional unit root exercise, the results of the current study show that the variables of interest are integrated of order one. Hence, the cointegration tests can be applied. The employed Bounds, Engle-Granger, and VAT cointegration tests concluded the existence of the long-run relationship among the variables. Based on the procedure proposed by Shafik and Bandyopadhyay (1992), it is concluded that the quadratic specification is the best fit relating the variables of interest, hence this functional form is used in the empirical estimations. Estimation results employing ARDL and FMOLS methods produced quite close results in terms of the magnitudes of the estimated coefficients. Examination of different variables in empirical estimations showed that *urbanization* and *administrative quality* variables do not have significant impact on *ecological footprint.* The impact of trade and energy consumption variables on ecological footprint is found to be positive and statistically significant. Based on estimation results, we can say that 1% increase in trade and energy consumption causes ecological footprint to increase by 0.17% (0.19% by FMOLS) and 0.55% (0.74% by FMOLS), respectively. The coefficient of energy consumption is somehow close to that of Ozturk et al. (2015), the only study investigating the same relationship in Azerbaijani case, being 0.83%. The slight difference might be due to the estimation period, being 1998–2008 in their case and 1996–2014 in our case. Ozturk et al. (2015) found negative coefficient for the trade variable, which does not seem proper considering the fact that in terms of consumption-based ecological footprint, Azerbaijan consumes more than its available biocapacity (Global Footprint Network 2018). Hence, our positive coefficient is in line with the expectations. The sign of the coefficient of the quadratic term (leading term) of income proxy is found to be positive, while the coefficient of linear term is negative. The positive sign of the leading term means that the relationship between income proxy and ecological footprint is U shaped. As the left wing of the U-shaped curve demonstrates the response of income from the beginning of the sample is decreasing, which does not seem to be reasonable, because as discussed in the related literature, the response is increasing first, and only after some threshold point can decline. Hence, we interpret a U shape as an evidence of N-shaped relationship as discussed in Lieb (2003), and therefore we conclude that relationship between ecological footprint and international tourism receipts is N shaped, which means currently the response of ecological degradation measure to income measure is positive and increasing. This conclusion is in line with the economic development stage of Azerbaijan. Ozturk et al. (2015) found the EKC in the case of Azerbaijan, which does not seem to be proper considering the country’s development path, and their finding might be due to the data sample which covers relatively ungrounded period of the development path.

In order to have the response of ecological footprint to income proxy from tourism sector, we should calculate the elasticity using the following formula:

2 $$ \mathrm{elasticity}={b}_1+2{b}_2\overline{y_t} $$

based on Eq. (1), where ${\overline{y}}_t$ is the logarithm of the mean of *international tourism receipts* (which is 15.72). Based on the estimated coefficients using ARDL, and employing the elasticity formula in (2), we found the elasticity to be 0.19. This means that 1% increase in income proxy results, on average, 0.19% increase in ecological footprint.

The use of VAT test also concluded the cointegration relationship between ecological footprint and income proxy, and the test for time-varying coefficient revealed that the coefficient of income variable is not time varying. Although the coefficient is found to be insignificant, its time profile provides some insights about the economy’s development trajectory as well as the examination of the EKC hypothesis.

From Fig. 3, where the varying elasticity is depicted versus per capita income level, we can derive the following conclusions: The income elasticity of ecological footprint increases, with some fluctuations, with the increase in income proxy, and after some point of income level it starts to increase steadily. The shape of the varying elasticity in Fig. 3 does not allow us to conclude the EKC phenomenon in the case of Azerbaijan, rather it says that the response of environmental degradation to increase in income proxied by international tourism receipts started to increase after some value of income. In addition, the shape of income elasticity depicted in Fig. 3, also confirms, the finding of U-shaped, consequently N-shaped relationship between the two variables of interest. Combining the findings of ARDL and TVC estimation results, we can say that the relationship between ecological footprint and income proxied by international tourism receipts does not confirm the EKC in Azerbaijan.

## Conclusion and policy implications

The study analyses the impact of tourism on environmental degradation, employing time-varying coefficient cointegration approach, which is the first application to the ecological footprint-tourism relationship, in addition to the conventional functional form, and fixed coefficient methods. After testing variables for unit root, the results showed their stationarity at first differenced form. Hence, the variables can be tested for a common long-run trend. The Bounds test, Engle-Granger test, and the VAT test for cointegration concluded a long-run co-movement among the variables. This implies that the variables, namely ecological footprint, energy consumption, and international tourism receipts share common trend in the long run. Results of the estimations, employing the conventional cubic functional form, revealed that the best fit for the relationship, in the case of Azerbaijan, is a quadratic functional form. We also tested explanatory variables such as urbanization, measures of administrative quality, energy consumption, and total trade. Except urbanization and measures of administrative quality, all other variables were found to have positive (sign wise) and statistically significant impact on ecological footprint. Furthermore, results of the empirical estimations, based on ARDL method, show that, numerically, 1% increase in trade and energy consumption causes ecological footprint to increase by 0.17% and 0.55%, respectively. Based on the results of the TVC approach, which takes into account the varying feature of the coefficient over time, the varying coefficient of income proxy was found to be insignificant, which means that the relationship can be modeled using the conventional approach. It is worth to mention that both conventional approach and the TVC method concluded the U-shaped relationship between ecological footprint and income (which can be interpreted as an N shaped, as discussed in Discussion section). To put differently, the results of the study show that between the variables of interest the EKC phenomenon does not hold in the case of Azerbaijan. The main findings and related policy implications of this study are as follows: (1) if the objective of the research is to conclude the nature of the relationship for specific case, and to make proper policy suggestions, then the relationship between environmental degradation and its drivers should be investigated individually for each country case, unless the purpose is to see the general (rough) tendency between the variables in a big picture; (2) if the conventional cubic specification is used, one should follow the proper methodology, namely starting with the cubic form, test for long-run co-movement to choose the best fit of the relationship and then continue from there; (3) the results of the fixed coefficient approach might be biased if the correct relationship is time varying. Since the coefficient is found to be time invariant, the relationship can be modeled employing the traditional approach. Numerically, based on the conventional approach utilizing the ARDL, the income elasticity of ecological footprint is found to be 0.19%; (4) the country’s policymakers should take into account the positive impact of trade, energy consumption, and income from tourism sector on environmental degradation, proxied by ecological footprint. In terms of negative impact (the sign is positive) of total trade on environmental degradation, the fact that Azerbaijan consumes significantly more than it possesses, in terms of available biocapacity (Global Footprint Network 2018), the structure of imported goods and services should be reconsidered. Regarding the positive impact of the energy consumption on environmental degradation, efficient energy use directions, activities to motivate public awareness in terms of adverse consequences of environmentally harmful energy use, and usage of alternative energy sources can be taken as relevant measures to follow. Taking into account the increasing interest and number of the international tourists to Azerbaijan, in order to mitigate the negative impact of the development of tourism sector on the environment and attain the macro-prudential and sustainable tourism development path, the set of regulations to be followed by the local service providers to the tourism sector, as well as the relevant control mechanism, need to be reconsidered and more environmental friendly measures should be taken.

As a direction for future research, the impact of international tourism on environmental degradation, proxied by CO_2_ emissions, needs to be studied for individual oil-exporting countries, considering the contributions of imports and exports separately.

## Footnotes

1 Based on one of the referee’s suggestion, we re-estimated the models with the transformed quarterly data and ended up with the similar results. Hence, we preferred to report the results with the actual data generating process, namely with annual data.

2 Based on one of the referee’s suggestion, we used real exchange rate as one of the potential drivers of environmental degradation (Katircioglu et al. 2018a, inter alia), but it is found to be insignificant.

## References

[CR1] Alam MS, Paramati SR (2016). The impact of tourism on income inequality in developing economies: does Kuznets curve hypothesis exist?. Ann Tour Res.

[CR2] Al-Mulali U, Fereidouni HG, Mohammed AH (2014a) The effect of tourism arrival on CO_2_ emissions from transportation sector. Anatolia: An International Journal of Tourism and Hospitality Research 26:230–243

[CR3] Al-mulali U, Fereidouni HG, Lee JMY, Mohammed AH (2014). Estimating the tourism-led growth hypothesis: a case study of the Middle East countries. Anatolia: An International Journal of Tourism and Hospitality Research.

[CR4] Anatasia V (2015). The causal relationship between GDP, exports, energy consumption, and CO2 in Thailand and Malaysia. Int J Econ Perspect.

[CR5] Apergis N (2016). Environmental Kuznets curves: new evidence on both panel and country-level CO_2_ emissions. Energy Econ.

[CR6] Azam M, Alam MM, Hafeez MH (2018). Effect of tourism on environmental pollution: further evidence from Malaysia, Singapore and Thailand. J Clean Prod.

[CR7] Balaguer J, Cantavella-Jorda M (2002). Tourism as a long-run economic growth factor: the Spanish case. Appl Econ.

[CR8] Becken S (2013). A review of tourism and climate change as an evolving knowledge domain. Tour Manage Perspect.

[CR9] Becken S, Hay J (2007). Tourism and climate change: risks and opportunities.

[CR10] Becken S, Frampton C, Simmons DG (2001). Energy consumption patterns in the accommodation sector-the New Zealand case. Ecol Econ.

[CR11] Becken S, Simmons DG, Frampton C (2003). Energy use associated with different travel choices. Tour Manag.

[CR12] Bouzahzah M, El Menyari Y (2013). International tourism and economic growth: the case of Morocco and Tunisia. J North Afr Stud.

[CR13] Bozkurt C, Akan Y, Okumus I (2016) Environmental Kuznets curve hypothesis in BRICTS: the role of tourısm. Economic and Social Development*:* Book of Proceedings, 59

[CR14] Brida JG, Cortes-Jimenez I, Pulina M (2016). Has the tourism-led growth hypothesis been validated? A literature review. Curr Issue Tour.

[CR15] Cetin M, Ecevit E (2017). The Impact of financial development on carbon emissions under the structural breaks: empirical evidence from Turkish economy. Int J Econ Perspect.

[CR16] Chang Y, Kim CK, Miller JI, Park JY, Park S (2014). Time-varying long-run income and output elasticities of electricity demand with an application to Korea. Energy Econ.

[CR17] Dawson J, Stewart EJ, Lemelin H, Scott D (2010). The carbon cost of polar bear viewing tourism in Churchill, Canada. J Sustain Tour.

[CR18] Dickey D, Fuller W (1981). Likelihood ratio statistics for autoregressive time series with a unit root. Econometrica.

[CR19] Dogan E (2017). CO2 emissions, real GDP, renewable energy and tourism: evidence from panel of the most-visited countries. Statistika-Stat Econ J.

[CR20] Dogan E, Seker F, Bulbul S (2015). Investigating the impacts of energy consumption, real GDP, tourism and trade on CO_2_ emissions by accounting for cross-sectional dependence: a panel study of OECD countries. Curr Issue Tour.

[CR21] Dritsakis N (2004). Tourism as a long-run economic growth factor: an empirical investigation for Greece using causality analysis. Tour Econ.

[CR22] Dubois G, Peeters P, Ceron JP, Gössling S (2011). The future tourism mobility of the world population: emission growth versus climate policy. Transp Res A.

[CR23] Durbarry R, Seetanah B (2015). The impact of long haul destinations on carbon emissions: the case of Mauritius. J Hosp Mark Manag.

[CR24] Engle RF, Granger CWJ (1987). Co-integration and error correction: representation, estimation and testing. Econometrica.

[CR25] Global Footprint Network (2018) National Footprint Accounts. https://www.footprintnetwork.org/resources/data/. Accessed on September 13, 2018

[CR26] Gössling S (2002). Global environmental consequences of tourism. Glob Environ Chang.

[CR27] Gössling S (2013). National emissions from tourism: an over looked policy challenge?. Energy Policy.

[CR28] Grossman GM, Krueger A (1993) Environmental impacts of a North American free trade agreement. In Barber P. (ed.), The US-Mexico Free Trade Agreement. MIT Press, Cambridge

[CR29] Grossman GM, Krueger A (1995). Economic growth and the environment. Q J Econ.

[CR30] Gunduz L, Hatemi-J A (2005). Is the tourism-led growth hypothesis valid for Turkey?. Appl Econ Lett.

[CR31] Hamilton JD (1994). Time series analysis.

[CR32] Hansen BE (1992). Efficient estimation and testing of cointegrating vectors in the presence of deterministic trends. J Econ.

[CR33] Hansen BE (1992). Tests for parameter instability in regressions with I(1) processes. J Bus Econ Stat.

[CR34] Hasanov FJ, Bulut C, Suleymanov E (2016) Do population age groups matter in the energy use of the oil-exporting countries? Econ Model (54):82–99

[CR35] Heidari H, Katircioglu ST, Saeidpour L (2015). Economic growth, CO2 emissions, and energy consumption in the five ASEAN countries. Int J Electr Power Energy Syst.

[CR36] Holden A (2009). The environment-tourism nexus influence of market ethics. Ann Tour Res.

[CR37] Howitt OJA, Revol VGN, Smith IJ (2010). Carbon emissions from international cruise ship passengers’ travel to and from New Zealand. Energy Policy.

[CR38] Hye QMA, Khan REA (2013). Tourism-led growth hypothesis: a case study of Pakistan. Asia Pac J Tour Res.

[CR39] Jaforullah M, King A (2017) The econometric consequences of an energy consumption variable in a model of CO2 emissions. Energy Econ 63: 84–91

[CR40] Jalil A, Mahmood T, Idrees M (2013). Tourism-growth nexus in Pakistan: evidence from ARDL bounds tests. Econ Model.

[CR41] Johansen S (1988). Statistical analysis of cointegration vectors. J Econ Dyn Control.

[CR42] Johansen S, Juselius K (1990). Maximum likelihood estimation and inference on cointegration with applications to the demand for money. Oxf Bull Econ Stat.

[CR43] Kalayci S, Koksal C (2015). The relationship between China’s airway freight in terms of carbon-dioxide emission and export volume. Int J Econ Perspect.

[CR44] Kapusuzoglu A (2014). Causality relationships between carbon dioxide emissions and economic growth: results from a multi-country study. Int J Econ Perspect.

[CR45] Katircioglu S (2009). Revisiting the tourism-led-growth hypothesis for Turkey using the bounds test and Johansen approach for cointegration. Tour Manag.

[CR46] Katircioglu S (2009). Trade, tourism and growth: the case of Cyprus. Appl Econ.

[CR47] Katircioglu S (2009). Testing the tourism-led growth hypothesis: the case of Malta. Acta Oeconomica.

[CR48] Katircioglu S (2010). International tourism, higher education, and economic growth: the case of North Cyprus. World Econ.

[CR49] Katircioglu ST (2011). Tourism and growth in Singapore: new extension from bounds test to level relationships and conditional Granger causality tests. Singap Econ Rev.

[CR50] Katircioglu ST (2014). Testing the tourism-induced EKC hypothesis: the case of Singapore. Econ Model.

[CR51] Katircioglu ST (2014). International tourism, energy consumption, and environmental pollution: the case of Turkey. Renew Sust Energ Rev.

[CR52] Katircioglu ST, Feridun M, Kilinc C (2014). Estimating tourism-induced energy consumption and CO_2_ emissions: the case of Cyprus. Renew Sust Energ Rev.

[CR53] Katircioglu ST, Fethi S, Kalmaz DB, Caglar D (2016) Interactions between energy consumption, international trade, and real income in Canada: an empirical investigation from a New Version of the solow growth model. Int J Green Energy 13(10):1059–1074

[CR54] Katircioglu S, Katircioglu S (2018) Testing the role of urban development in the conventional environmental Kuznets curve: evidence from Turkey. Appl Econ Lett 25(11):741–746 10.1007/s11356-018-1651-9 29552719

[CR55] Katircioglu S, Gokmenoglu K, Eren B (2018). Testing the role of tourism development in ecological footprint quality: evidence from top 10 tourist destinations. Environ Sci Pollut Res.

[CR56] Katircioglu S, Katircioglu ST, Altinay M (2018). Interactions between tourism development and financial development. Serv Ind J.

[CR57] Katircioglu S, Katircioglu ST, Kilinc CC (2018c) Investigating the role of urban development in the conventional environmental Kuznets curve: evidence from the globe. Environ Sci Pollut Res 25(15):15029–15035 10.1007/s11356-018-1651-9 29552719

[CR58] Kilinc CC, Semiz M, Katircioglu E, Unusan C (2013). Choosing restaurant for lunch in campus area by the compromise decision via AHP. Int J Econ Perspect.

[CR59] Kuznets S (1955). Economic growth and income inequality. Am Econ Rev.

[CR60] Lee JW, Brahmasrene T (2013). Investigating the influence of tourism on economic growth and carbon emissions: evidence from panel analysis of the European Union. Tour Manag.

[CR61] León CJ, Arana JE, Alemán AH (2014). CO_2_ emissions and tourism in developed and less developed countries. Appl Econ Lett.

[CR62] Liddle B, Messinis G (2016) Revisiting carbon Kuznets curves with endogenous breaks modeling: evidence of decoupling and saturation (but few inverted-us) for individual OECD countries. Empir Econ. 10.1007/s00181-016-1209-y

[CR63] Lieb CM (2003) The environmental Kuznets curve – a survey of the empirical evidence and of possible causes. University of Heidelberg Discussion Paper No. 391

[CR64] Mackinnon JG (1996) Numerical distribution functions for unit root and cointegration test. J Appl Econ 11:601–618

[CR65] Mikayilov JI, Shukurov V, Mukhtarov S, Yusifov S (2017). Does urbanization boost pollution from transport?. Acta Universitatis Agriculturae et Silviculturae Mendelianae Brunensis.

[CR66] Mikayilov J, Shukurov V, Yusifov S (2017). The impact of economic growth and population on CO_2_ emissions from transport sector: Azerbaijan case. Acad J Econ Stud.

[CR67] Mikayilov JI, Galeotti M, Hasanov FJ (2018). The impact of economic growth on CO_2_ emissions in Azerbaijan. J Clean Prod.

[CR68] Mikayilov JI, Hasanov FJ, Galeotti M (2018). Decoupling of CO_2_ emissions and GDP: a time-varying cointegration approach. Ecol Indic.

[CR69] Mikayilov JI, Apergis N, Hasanov FJ (2018c) CO_2_ emissions-economic growth relationship revisited: new insights from the time-varying cointegration approach. Unpublished work

[CR70] Moosa IA (2017) The econometrics of the environmental Kuznets curve: an illustration using Australian CO_2_ emissions. Appl Econ 49:4927–4945. 10.1080/00036846.2017.1296552

[CR71] Mukhtarov S, Mikayilov JI, Ismayilov V (2017). The relationship between energy consumption and economic growth: evidence from Azerbaijan. Int J Energy Econ Policy.

[CR72] Mukhtarov S, Mikayilov JI, Mammadov J, Mammadov E (2018). The impact of financial development on energy consumption: evidence from an oil-rich economy. Energies.

[CR73] Naradda Gamage SK, Hewa Kuruppuge R, Haq IU (2017). Energy consumption, tourism development, and environmental degradation in Sri Lanka. Energy Sour B Econ Plan Pol.

[CR74] Narayan PK (2005). The saving and investment nexus for China: evidence from cointegration tests. Appl Econ.

[CR75] Narayan PK, Prasad BC (2003). Does tourism granger causes economic growth in Fiji?. Empir Econ Lett.

[CR76] Nepal SK (2008). Tourism-induced rural energy consumption in the Annapurna region of Nepal. Tour Manag.

[CR77] Neto F (2003). A new approach to sustainable tourism development: moving beyond environmental protection. Nat Res Forum.

[CR78] Ozturk I, Al-Mulali U, Saboori B (2015) Investigating the environmental Kuznets curve hypothesis: the role of tourism and ecological footprint. Environ Sci Pollut Res 23(2):1916–1928 10.1007/s11356-015-5447-x 26408117

[CR79] Paramati SR, Shahbaz M, Alam MS (2017). Does tourism degrade environmental quality? A comparative study of eastern and Western European Union. Transp Res D.

[CR80] Paramati SR, Alam MS, Chen CF (2017b) The effects of tourism on economic growth and CO_2_ emissions a comparison between developed and developing economies. J Travel Res:1–13. 10.1177/0047287516667848

[CR81] Park JY, Rhodes GF, Fomby TB (1990). Testing for unit roots and cointegration by variable addition. Advances in econometrics.

[CR82] Park JY (1992). Canonical cointegrating regressions. Econometrica.

[CR83] Park JY, Hahn SB (1999). Cointegrating regressions with time varying coefficients. Econ Theory.

[CR84] Perch-Nielsen S, Sesartic A, Stucki M (2010). The greenhouse gas intensity of the tourism sector: the case of Switzerland. Environ Sci Pol.

[CR85] Pesaran M, Shin Y, Strom S (1999). An autoregressive distributed lag modeling approach to cointegration analysis. Econometrics and economic theory in the 20th century: the Ragnar Frisch centennial symposium.

[CR86] Pesaran MH, Shin Y, Smith RJ (2001). Bounds testing approaches to the analysis of level relationships. J Appl Econ.

[CR87] Phillips PCB, Hansen BE (1990). Statistical inference in instrumental variables regression with I(1) processes. Rev Econ Stud.

[CR88] Phillips PB, Perron P (1988). Testing for unit roots in time series regression. Biometrika.

[CR89] Raza SA, Sharif A, Wong WK, Karim MZA (2016) Tourism development and environmental degradation in the United States: evidence from wavelet-based analysis. Curr Issues Tour:1–23. 10.1080/13683500.2016.1192587

[CR90] Rosselló-Batle B, Moià A, Cladera A, Martínez V (2010). Energy use, CO_2_ emissions and waste throughout the life cycle of a sample of hotels in the Balearic Islands. Energy Build.

[CR91] Saenz-de-Miera O, Rosselló J (2014). Modeling tourism impacts on air pollution: the case study of PM10 in Mallorca. Tour Manag.

[CR92] Saikkonen P (1992). Estimation and testing of cointegrated systems by an autoregressive approximation. Econ Theory.

[CR93] Shafik N, Bandyopadhyay S (1992). Economic growth and environmental quality: time series and cross-country evidence.

[CR94] Shakouri B, Khoshnevis Yazdi S, Ghorchebigi E (2017). Does tourism development promote CO2 emissions?. Anatolia.

[CR95] Solarin SA (2014). Tourist arrivals and macroeconomic determinants of CO2 emissions in Malaysia. Anatolia.

[CR96] Stock JH, Watson M (1993). A simple estimator of cointegrating vectors in higher order integrated systems. Econometrica.

[CR97] Strategic Road Map for the Development of Specialized Tourism Industry in Azerbaijan (2016) https://azertag.az/en/xeber/Azerbaijan_endorses_strategic_road_maps_for_development_of_national_economy_and_main_economic_sectors-1016958. Accessed on September 13, 2018

[CR98] Tang CF, Abosedra S (2014). Small sample evidence on the tourism-led growth hypothesis in Lebanon. Curr Issue Tour.

[CR99] Tang CF, Abosedra S (2014). The impacts of tourism, energy consumption and political instability on economic growth in the MENA countries. Energy Policy.

[CR100] Tang C, Tan E (2015). Does tourism effectively stimulate Malaysia’s economic growth?. Tour Manag.

[CR101] Tovar C, Lockwood M (2008). Social impacts of tourism: an Australian regional case study. Int J Tour Res.

[CR102] Tsagarakis K, Bounialetou F, Gillas K, Profylienou M, Pollaki A, Zografakis N (2011). Tourist’s attitudes for selecting accommodation with investments in renewable energy and energy saving systems. Renew Sust Energ Rev.

[CR103] Tsai KT, Lin TB, Hwang RL, Huang YJ (2014). Carbon dioxide emissions generated by energy consumption of hotels and homestay facilities in Taiwan. Tour Manag.

[CR104] UNEP (United Nations Environment Programme) (2005) http://www.unep.fr/shared/publications/pdf/dtix0592xpa-tourismpolicyen.pdf. Accessed 17 April 2019

[CR105] UNWTO (United Nations World Tourism Organization) (2017) Tourism highlights, 20147 edn

[CR106] Vita GD, Katircioglu S, Altinay L, Fethi S, Mercan M (2015). Revisiting the environmental Kuznets curve hypothesis in a tourism development context. Environ Sci Pollut Res.

[CR107] World Bank (1992). The world development report 1992.

[CR108] World Bank (2018a) World development indicators. http://databank.worldbank.org/data. Accessed on June 12, 2018

[CR109] World Bank (2018b) The worldwide governance indicator (WGI). http://info.worldbank.org/governance/wgi/index.aspx#home. Accessed on July 01, 2018

[CR110] Xuchao W, Priyadarsini R, Eang LS (2010). Benchmarking energy use and greenhouse gas emissions in Singapore’s hotel industry. Energy Policy.

[CR111] Yorucu V (2016). Growth impact of CO_2_ emissions caused by tourist arrivals in Turkey: an econometric approach. Int J Clim Chang Strateg Manag.

[CR112] Zaman K, Shahbaz M, Loganathan N, Raza SA (2016). Tourism development, energy consumption and environmental Kuznets curve: trivariate analysis in the panel of developed and developing countries. Tour Manag.

[CR113] Zhang L, Gao J (2016). Exploring the effects of international tourism on China’s economic growth, energy consumption and environmental pollution: evidence from a regional panel analysis. Renew Sust Energ Rev.

